# Peripheral Arterial Disease Study (PERART): Prevalence and predictive values of asymptomatic peripheral arterial occlusive disease related to cardiovascular morbidity and mortality

**DOI:** 10.1186/1471-2458-7-348

**Published:** 2007-12-11

**Authors:** María Teresa Alzamora, José Miguel Baena-Díez, Marta Sorribes, Rosa Forés, Pere Toran, Marisa Vicheto, Guillem Pera, María Dolores Reina, Carlos Albaladejo, Judith Llussà, Magda Bundó, Amparo Sancho, Antonio Heras, Joan Rubiés, Juan Francisco Arenillas, the PERART study

**Affiliations:** 1 Primary Healthcare Centre Riu Nord-Riu Sud, Institut Català de la Salut, Santa Coloma de Gramenet,, Spain; 2 Department of Medicine, Universitat Autònoma de Barcelona, Spain; 3 Research Unit Barcelonés Nord Maresme. ICS-IDIAP Jordi Gol. Mataró, Spain; 4 Primary Healthcare Centre La Marina, Institut Català de la Salut, Barcelona, Spain; 5 Primary Healthcare Centre Numància, Institut Català de la Salut, Barcelona, Spain; 6 Primary Healthcare Centre Santa Coloma de Gramenet, Institut Català de la Salut, Santa Coloma de Gramenet, Spain; 7 Primary Healthcare Centre Llefià, Institut Català de la Salut Badalona, Spain; 8 Primary Healthcare Centre Sant Roc, Institut Català de la Salut, Badalona, Spain; 9 Primary Healthcare Centre Ronda Prim, Institut Català de la Salut, Mataró, Spain; 10 Primary Healthcare Centre Can Mariné, Institut Català de la Salut, Santa Coloma de Gramenet, Spain; 11 Unitat de Neurociències. Hospital Germans Trias i Pujol. Institut Català de la Salut. Badalona, Spain

## Abstract

**Background:**

The early diagnosis of atherosclerotic disease is essential for developing preventive strategies in populations at high risk and acting when the disease is still asymptomatic. A low ankle-arm index (AAI) is a good marker of vascular events and may be diminished without presenting symptomatology (silent peripheral arterial disease). The aim of the PERART study (PERipheral ARTerial disease) is to determine the prevalence of peripheral arterial disease (both silent and symptomatic) in a general population of both sexes and determine its predictive value related to morbimortality (cohort study).

**Methods/Design:**

This cross-over, cohort study consists of 2 phases: firstly a descriptive, transversal cross-over study to determine the prevalence of peripheral arterial disease, and secondly, a cohort study to evaluate the predictive value of AAI in relation to cardiovascular morbimortality.

From September 2006 to June 2007, a total of 3,010 patients over the age of 50 years will be randomly selected from a population adscribed to 24 healthcare centres in the province of Barcelona (Spain).

The diagnostic criteria of peripheral arterial disease will be considered as an AAI < 0.90, determined by portable Doppler (8 Mhz probe) measured twice by trained personnel. Cardiovascular risk will be calculated with the Framingham-Wilson tables, with Framingham calibrated by the REGICOR and SCORE groups. The subjects included will be evaluted every 6 months by telephone interview and the clnical history and death registries will be reviewed. The appearance of the following cardiovascular events will be considered as variables of response: transitory ischaemic accident, ictus, angina, myocardial infartction, symptomatic abdominal aneurysm and vascular mortality.

**Discussion:**

In this study we hope to determine the prevalence of peripheral arterial disease, especially the silent forms, in the general population and establish its relationship with cardiovascular morbimortality. A low AAI may be a better marker of arterial disease than the classical cardiovascular risk factors and may, therefore, contribute to improving the predictive value of the equations of cardiovascular risk and thereby allowing optimisation of multifactorial treatment of atherosclerotic disease.

## Background

Prevention and early diagnosis of atheroslerotic disease is one of the essential objectives of the field of cardiovascular disease since it is the main cause of mortality in developed countries. In Spain, cardiovascular diseases are the first cause of death, producing 34% of all deaths [1], with ischaemic heart disease causing the greatest number of cardiovascular deaths (31%) followed by cerebrovascular disease (29%) [1].

Atherosclerosis is currently considered a chronic, progressive, systemic disease, the genesis of which involves complicated pathogenic mechanisms. This disease is characterised by remaining clinically silent during most of its evolutive process until the sudden appearance of complications of the atherosclerotic plaques which lead to ischaemic vascular events. From primary prevention point of view, it is therefore very important to develop strategies to identify patients with subclinical stages of arteriosclerosis.

Atherosclerotic disease is characterised by a multifactorial aetiology constituted by different risk factors which potentiate among themselves and which are often presented in asssociation [2]. Risk factors which may be modified include arterial hypertension, hypercholesterolemia, diabetes mellitus, and smoking, while age and sex are non modifiable factors. Other risk factors act through intermediate risk factors such as abdominal obesity [3,4], sedentarism, or a family history of early coronary heart disease. These risk factors have been integrated in prediction tables based on regression models with the aim of detecting the population at high risk of presenting cardiovascular events [5]. However, the sensitivity and positive predictive value of these tables is low and, thus, most cardiovascular diseases are produced in persons without a high risk [5]. It is, therefore, important to develop markers of silent ateriosclerotic disease which may aid to better define the patients at high risk and to thereby act early and intensively to prevent the appearance of cardiovascular diseases with pharmacologic and non pharmacologic measures.

The ankle-arm index (AAI) is the result of the quotient obtained by dividing systolic arterial pressure (SAP) at the level of the posterior pedial or tibial artery between the brachial SAP by Doppler [6]. Low values of AAI determination (<0.9) allow the diagnosis of peripheral arterial disease (PAD) in asymptomatic patients [7]. An AAI of < 0.9 has a sensitivity of 95% and a specificity of 99% for angiographically demonstrated PAD [8]. Determination of the AAI also allows the quantification of the severity of PAD: mild (AAI = 0.7 – 0.9), moderate (AAI = 0.5 – 0.69) and severe stenosis (AAI = <0.5).

In some studies a low AAI has shown to be a good marker of vascular events [8]. In the Atherosclerosis Risk in Communities (ARIC) Study [9,10], the AAI was inversely associated with coronary disease, ictus and atherosclerosis at the level of the carotid and popliteal arteries. The Edinburgh Artery Study [11] reported a relative risk of ictus of 1.98 (CI 95%; 1.05 – 3.77) in patients with an AAI < 0.9. In a Swedish cohort of males over the age of 68 years [12] followed over 10 years, the relative risk of ischaemic ictus for an AAI = 0.9 was 2.0 (95% CI; 1.1 – 3.7). These results, however, do not agree in all studies. In the Cardiovascular Health Study [7], an assocation was found between a low AAI and the risk of vascular events in general, but no relationship was observed between an AAI < 0.9 and the risk of ictus. In addition, in the Pittsburgh study the risk of cardiovasscular events significantly increased only above an AAI < 0.7 [13].

Another aspect requiring clarification is the prognostic value of AAI in a medium with a low prevalence of cardiovascular diseases such as in Spain and other Mediterranean countries [14].

Moreover, the PAD is a significant predictor of cardiovascular mortality [15-17]. Mortality is also greater in patients with asymptomatic PAD compared to patients without arterial disease [16,18,19]. Some studies [7] have suggested that PAD in females represents a greater cardiovascular morbidity while in males this disease represents a higher mortality rate.

The prevalence of PAD ranges between 1.8% and 25% according to the population, the medium studied and the cut-off value of the AAI [11,20-25]. Likewise, the prevalence is higher in determined populational subgroups such as diabetics [20] and smokers [23]. No populational based studies are available in Spain, with the studies found being carried out in selected populations [26-34].

The present study will have two phases. The main objective of the first phase (cross-over study) is to determine the prevalence of PAD in the general population, in both its silent and symptomatic forms. The secondary objective is to study the association of PAD, especially in the silent forms, with other prevalent cardiovascular events and cardiovascular risk factors. The main objective of the second phase (cohort study) is to determine the incidence of cardiovascular events in patients with PAD and quantify the risk of having these events. The secondary objective in this phase is to study the predictive value of AAI with respect to the risk of having cardiovascular events in relation to the equations of cardiovascular risk (Framingham-Wilson, Framingham calibrated by REGICOR and SCORE).

## Methods/Design

This is a two-phase study. The first phase is a transversal, multicentre, descriptive study the aim of which is to determine the prevalence of symptomatic and asymptomatic PAD in a general population attended in primary care. The secondary aim is to study the association of PAD, especially in the silient forms, with other previous or prevalent cardiovascular events and cardiovascular risk factors. The second phase is a cohort study, the main objectives of which are to determine the incidence of cardiovascular events in patients with and without PAD and to quantify the risk of having these events. The secondary objective is to study the predictive value of AAI with respect to the risk of having cardiovascular events in relation to the equations of cardiovascular risk (Framingham, Framinham calibrated by REGICOR and SCORE). An initial 5-year follow up will be carried out in the cohorts generated in phase I: subjects without PAD and subjects with PAD and the cardiovascular morbimortality will be analysed. This study has been approved by the local Ethics Committee (Jordi Gol i Gurina Foundation of Investigation in Primary Care). Informed written consent will be obtained from all the participants. Likewise, the recommendations of the World Medical Assocation Declaration of Helsinki will be followed [35].

### Setting

This study will be performed in a total of 24 healthcare centres within the metropolitan area of the city of Barcelona and the county of North Barcelona – Maresme, including urban and semi-rural healthcare centres. These centres cover a population of approximately 600,000 inhabitants.

### Study Population and subjects

**Figure 1:**
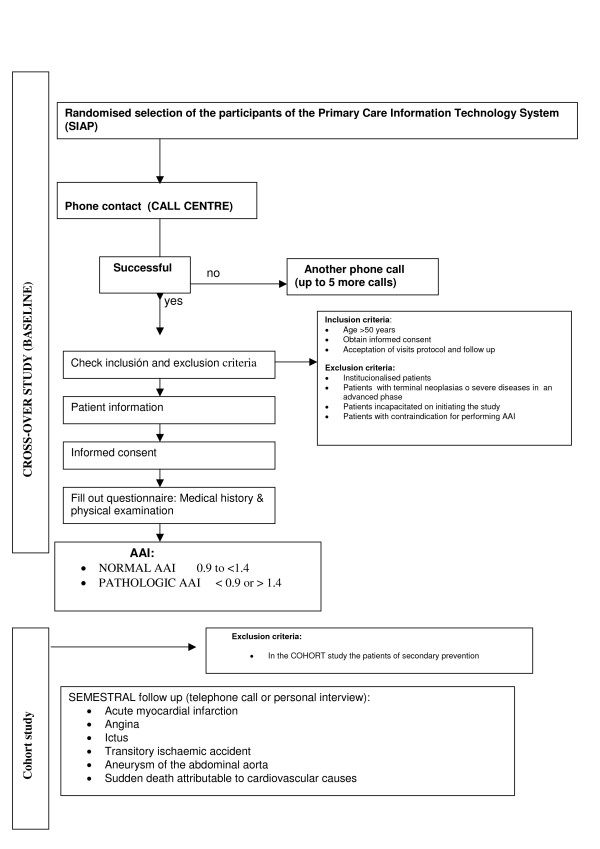
Flow diagram of the study.

A simple randomised sample will be undertaken until achieving the required sample size. Figure 1 provides a flow diagram of the study.

### Data collection

Data will be collected in a questionnaire specifically designed for this study. Prior to initiation of the study a pilot trial will be performed in 120 cases to detect errors in its design, to test the questionnaire interviewers and to verify concordance in data collection (data validation).

#### Baseline data collections (cross-over study)

The following variables will be studied.

-Demographic, cultural and economic variables related to the capacity of physical strength, with a previously validated questionnaire [36], related to menopause, family history of early cardiovascular death, personal history of cardiovascular disease, acute myocardial infarction, angina, cerebrovascular accident [14]. Similarly, the clinical history will be verified if the patient is diagnosed with ictus (CIE 10 code: I64), transitory ischaemic accidents (CIE 10 code: G45), angina (CIE 10 code: I20.0), acute myocardial infarction (CIE 10 code: I25) and PAD of the lower extremities (CIE 10 code: I73.9). Personal history of hypercholesterolemia, diabetes mellitus, arterial hypertension and smoking, establishing the history and current status of the smoking habit in the latter case [14,37-39].

-Anthropometric variables, current systolic/diastolic blood pressure values, blood analysis (total cholesterol, HDL-cholesterol, DLD-cholesterol, triglycerides and glycaemia [glycosylated haemoglobin (HbA1c) in diabetics], except in the case of having a recent analysis within the last 12 months, and standard electrocardiogramme [40]. Calculation of cardiovascular risk with the Framingham-Wilson equations [40], Framingham calibrated by the REGICOR [41] and SCORE [42] groups, and medications taken considering current drug intake (at least 1 tablet/day) of the following pharmacological groups: lipid-lowering drugs, antiaggregants, anticoagulants, antihypertensives and hypoglycidemiants [43,44]; Edinburgh vascular questionnaire [45].

The ankle-arm index (AAI) examination of the subjects will be carried out in the 24 healthcare centres participating in the study by two healthcare professionals trained in the technique. The AAI will be performed in the two paramaleolar arteries of both lower extremities, taking into consideration the lowest of four AAI determinations. If the result is < 0.9, the technique will be performed by the other professional. In cases in which the second professional finds an AAI>= 0.9, the first will repeat the test and the latter value will be considered as the final result. AAI measurement will be performed by the standardised procedure [6].

Patients with peripheral arterial disease will be referred to the general practitioner of the healthcare centre to which the patient is adscribed to follow the protocols of the centre. If the AAI is lower than 0.5, the patient will be referred to the vascular surgeon of reference.

#### Follow up (cohort study)

After determination of the AAI the subject will enter in one of the cohorts (AAI>= 0.9: cohort without peripheral arterial disease; AAI < 0.9 cohort with peripheral arterial disease).

The successive controls will be undertaken every 6 months after the last visit, by telephone interview. The appearance of any of the events will be registered in the data collection protocol: transitory ischaemic accident, ictus, angina, myocardial infarction, symptomatic abdominal aneurysm and mortality attributable to vascular causes.

The main variable of response will be the appearance of cardiovascular events as a combined compound variable [27] made up of the following diseases:

#### Morbidity

-Coronary disease: 1) angina, 2) acute myocardial infarction

-Cerebrovascular disease: 1) transient ischaemic attack, 2) ictus

-Symptomatic aneurysm of the abdominal aorta

These events will be confirmed by a cardiologist, neurologist and vascular surgeon, respectively.

#### Mortality

-Ictus

-Myocardial infarction

-Sudden death attributable to cardiovascular events

-Aneurysm of the abdominal artery

-Complications of PAD.

The mortality will be studied by: control of the computerised clinical history, telephone interview with a relative and personal or telephone interview with general practitioner responsible for the patient. If adequate information is not obtained by the above sources, the emergency departments (SEM) and emergency paramedical services (061) and, if necessary, the statistical registries of deaths (Department of Health) will be consulted.

### Statistical analysis

#### Sample size

The sample size for the cohort study has been calculated with an alpha risk of 0.05, a beta risk of 0.20 in a bilateral contrast, taking a relationship of exposure (altered AAI) compared to no exposure (normal AAI) of 1:9 into account. To do this 301 subjects in the group of patients diagnosed with peripheral arterial disease (pathologic AAI) and 2709 patients in the group without peripheral arterial disease (normal AAI) are required to detect a minimum relative risk of the main variable of response of 2, accepting an incidence of the cardiovascular disease studied in the general population of 5.2% [24]. The rate of loss to follow up has been calculated to be of 10%.

This sample size also ensures correct estimation of the populational prevalence of asymptomatic AAI in our medium with a precision of 2%, accepting an alpha risk of 0.05 in the most unfavourable case of having to detect a prevalence of 50%.

#### Data analysis

This will be performed with the Stata 9 statistical package after the introduction of the data in a database (Acces).

Quantitative variables will be compared with the Student's t test and analysis of variance (ANOVA) will be performed, using the corresponding non parametric tests when necessary. Qualitative variables comparisons will be determined using the Chi squared test.

An analysis of survival of each vascular event will be performed according to the presence/absence of PAD with the Kaplan-Meier curves. Cox multivariate regression models will be used to compare the probability of having a cardiovascular event in the follow up cohorts, adjusting for the necessary covariables. The relative risk (hazards ratio) will be analysed with a confidence interval of 95%.

## Discussion

The aim of the PERART study is to evaluate the real prevalence of peripherial arterial disease in the general population which remains to be determined in Spain and is poorly known in countries with a low cardiovascular risk. These data will be the main contribution of this study since, to date only data of series of patients and very selected populations have been established.

With the present study we hope to demonstrate that the silent forms of peripheral arterial disease may be a marker of cardiovascular risk with a better adjustment than the methods based on risk tables since these are indicative of real damage in the cardiovascular system still in presymptomatic stages.

It is therefore important to keep in mind that the AAI may have a predictive value which is higher than the traditional methods of calculating cardiovascular risk since these methods are based on probabilistic mathematical equations while the AAI detects early stages of atherosclerotic disease. On being a systemic disease, the detection of peripheral involvement is probably indicative of asymptomatic involvement of other vascular territories, and thus, the determination of AAI may have diagnostic and prognostic significance in the detection of asymptomatic cerebrovascular disease. Another interesting aspect is the follow up of the patients with a high AAI, which may also indicate a certain risk of having cardiovascular events if associated with arterial calcification [32].

If the results confirm our hypothesis we will have an easy, accessible, inexpensive test, which is reproducible in Primary Care and which will allow a better selection of patients in whom an approach of greater therapeutic intensity may be applied to thereby avoid or slow major cardiovascular complications and events. We may then have a test to allow screening for selecting patients prior to performing more complex tests or to decide whether to intensify the interventions in lifestyle or with drugs (statins, acetylsalicillic acid, etc.)

On the other hand, the populational, multicentric, prospective design of the study, which collects most of the cardiovascular risk factors will allow our cohort to be used to verify the validity of the existing cardiovascular risk factors or those of other similar future projects within the age range of this study.

The PERART study may have certain limitations such as not including a population of under the age of 50 years because of the high prevalence of PAD in this age group. Likewise, according to the different studies the variability of the calculation of the AAI between previously trained observers is 7% [46]. To reduce the interobserver variability to a minumun, the AAI will only be performed by two specially trained healthcare professionals.

The sensitivity of the AAI as a predictor of vascular events is low (compared to the specificity). Nonetheless, it will probably be greater than the traditional methods of calculating cardiovascular risk. The Framingham tables and REGICOR are designed to calculate cardiovascular risk in patients between 35 and 74 years in age. In patients > 74 years we will calculate the risk assigning the score corresponding to 74 years (maximum risk for age). On the other hand, we will be working with symptomatic diseases and thus the patients will be receiving medication, which may modify the natural history of the disease. However, it would not be ethical to discontinue antiaggregant treatment in subjects with a diminished AAI. This means that any effect of PAD with respect to the appearance of cardiovascular events will be lower than that provoked by PAD left to its natural evolution without diagnosis or treatment.

In conclusion, the PERART study will attempt to determine the prevalence of PAD in a low cardiovascular risk, Spanish population, and especially, to determine the predictive value of pathologic AAI in relation to the risk of having cardiovascular disease since it may be a better marker than the classical cardiovascular risk factors because of its being indicative of the presence of arterial lesion.

## Competing interests

The author(s) declare that they have no competing interests.

## Authors' contributions

MTA, JMB, MS, RF, PT, MV, MDR, JL and MB participated in the design of the study;

MTA, JMB, MS, RF, PT, CA, JL and AS contributed to the coordination study; GP participated in the statistical calculations.

All the authors have read and approved the final manuscript.

## Pre-publication history

The pre-publication history for this paper can be accessed here:


